# The Dental Pulp and Pulpless Teeth

**Published:** 1875-06

**Authors:** Milton H. Chappell

**Affiliations:** Knightstown, Ind.


### THE DENTAL PULP AND PULPLESS TEETH.

BY MILTON H. CHAPPELL, KNIGHTSTOWN, IND.

*Read before the Eastern Indiana Dental Association, May 4th*, 1875.

In dental practice, the cases of pulp exposures with phase and stage of disease,—pulpless teeth with discolored enamel, periostitis, and alveolar abscess are frequently the source of unexpected disappointment.

The dental pulp exposed by caries and aching gives us the cases the most difficult to treat, owing to our limited ability to properly determine the extent of the disease of the tissues involved of which we may be deceived,—the structure may be slowly passing away by suppuration, or by the constant irritation nature is sending her force to heal the defect. Granules or calcification takes possession of the pulp chamber, and a variety of evils afflict our patient. Some such cases give no trouble or warning of their demise, but they are the exception. Then we have the slight exposures, even the removed of the enamel, exposing the nerve fibrils, causing pain and the dentine appears inflamed, such cases present great vitality of nerve tissue and susceptibility when the pulp-tissue (capillary systemic circulation) is exposed, we may expect a natural remedy—secondary dentine; when abused granules or calcification the repair of such tissues is by adhesive, or health restoring inflammation, necessary for the deposit of dentine. We must respect these pathological conditions in the treatment of pulps or our success is uncertain. with patients in good health, free from climatic or miasmatic derangement, the treatment is simple, apply the dam, cleanse thoroughly, and if there be pain without exposure of the pulp membranes you must judge by the length and severity of the pain the character of the disease, apply palliative agents, if acute or primary inflammation it will subside in a short time, of its own recuperative energy, apply dry oxide zinc, lay a piece of spongoid in cavity, rest a few moments, if easy, apply a shaving of Hill's Stopping lined with arnica plaster, made by laying the shaving on the handle of a separating file, cut the plaster, the size desired and lay on the shaving place over spirit lamp press together, moisten slightly the plaster and lay over the pulp region as a protection from thermal changes fill over with oxychloride of zinc, it is better in all such cases to not fill with gold at the first sitting, wait several days. Where there is continued pain in cleansing and diagnosing apply a palliative, or rupture the pulp membrane and taking away several drops of blood will be very satisfactory, if there has been any pus cells or granules forming they will pop away by the depletion and healthy blood plasm will supply the need and a speedy return to health. When the hemorrhage and pain have ceased apply glycerine and oxide zinc then the plaster and Hill's Stopping as before mentioned and subsequently filling the same provided the depletion does not carry away the pus cells, or the part nearest the exposure may be in a state of suppuration. Remove the disease by an operation if possible; if there is ever any excuse for the use of escharotics it is in these cases. Apply a solution of creosote and dry oxide zinc, a layer of lint of spongoid to absorb the exudation, fill over with wax or Hills Stopping for one or two days, dress the wound the same, as soon as health is restored, apply the plaster and Hill's Stopping filling with oxy-chloride after several weeks, if all is quiet examine carefully for the life of the tooth; cut out sufficiently and fill permanently.

If the pulp is exposed by accident and all parts healthy dress with glycerine and tannin if continued hemorrhage, otherwise apply the plaster cap, which is free from irritation, and Hill's Stopping etc., as before mentioned. Fill permanently at first sitting if desired to complete—the case will be all right. In patients with colds, anæmia or scorbutic, the treatment of tooth pulp is attended with much uncertainty, unless the treatment is prolonged and manipulation careful. Respecting the idiosyncrasy of the case—if there is any excuse for using escharotics (creosote or carbolic acid) it is in these cases, but I believe it should not be used unless there is suppuration. I am convinced by the failures in treating pulps that the greatest number of failures is by this indiscriminate use of creosote and oxy-coloride for healthy simple exposures. Doctor the pulp if healthy, as little as possible. The great Master will, if possible heal the affliction if given a chance by natural means, through the function of the blood. The continued contact of escharotics or carious substance will cause the formation of granules or calcification (of which I have a number of specimens) or disorganization, suppuration, periostitis and finally alveolar abscess. The pulp being so vascular and peculiarly sensitive we must avoid and protect it from such irritating agents.

When my patients are willing to assist me in the proper care of their teeth and I find a pulp so far disorganized that its room is better than its company; I treat it as it deseaves, (avoiding the dangerous effects of arsenic, and why do we always use creosote with arsenic in the paste?) Apply carbolic acid, seal up securely and await my time, examine and repeat the application if necessary until you get to the apex of the root, where the life forces of the periosteum lends a helping hand and holds the enemy in check by the narrow gate way but if arsenic is used, bone does not seem any barrier and periostitis too often supervenes.

There are two kinds to be considered, and in the mouths of persons as variously constituted as in the treatment of the pulp, holding the same systemic conditions as my true guide avoiding hastily permanent work, with anemic constitutions and persons with climatic oppression until more favorable state of affairs presents. In cases where health abounds and recent removal of the pulp membranes the treatment is simple. Have thoroughly dry, measure the depth of canal and fill with gold or very dry oxy-chloride if the apex be small to the entire depth, the removal of the membrane from the buccal superior molar roots is puzzling and at times impossible of both canals. My choice is glycerine or tannin, do the best you can in removing, hoping the tannin has withered the remaining portions. Closing the foramen preventing a reservoir for liquids. The palatine root being direct and generally very large, can and should be treated every timeand filled to apex. The lower molars treat as the upper molars trusting on the tannin where you can not do better. In all the other teeth you have free access to clear the canals, avoiding reservoirs and filling to apex of root.

In cases where pulps are dead, and abscesses formed, pus discharging through canal, sometimes abscess through the alveolus, the pus formation and flow. Cleanse by operation through the canal, open with Gage's drills to abscess sac, if no other opening for discharge; but if abscess discharge through the gum, open thoroughly to point of thc sac, form a communication through the tooth, using topically sol. carbolic acid, if not yeilding readily, adding tr. iodine and glycerine. Closing the canal to apex with Hill's Stopping or oxychloride and treat through the gum, if there is no abscess through the gum, form a piston of Hill's Stopping and force the remedy through to the sac, allow to remain short time, and dress by placing floss silk in canal, when you discover no taint of pus or caries fill temporarily, wait a reasonable time then fill permanently. When we have periostitis, the application of tr. iodine over the gum will give relief, sometimes a cantharidal blister will be necessary, the administration of a mild cathartic or attentive will work wonders in the treatment of pulpless teeth, my choice is Dr. Chase's specific enlarged. Hyg., Sub. Mur. v grs; sugar milk xl grs., thorougly triturated, one grain doses until relief which in many cases acts like a charm if such a thing could be so considered.

If systemic conditions in the treatment of dental pulps, and pulpless teeth are not recognizèd, we have an old brooding hen on our hands. The general health, climatic and misasmatic changes, have much to do in treating the same. Since 1866 I kept a correct register of all operations, and for three years I have adopted the present mode, treating pulps and pulpless teeth, noting the exact condition, and treatment, and datc of subsequent report; whether any trouble, complete success or failure. I feel justified in continuing this mode for the preservation of "the dental pulps, and pulpless teeth."

